# Preoperative and postoperative administration of vitamin C in cardiac surgery patients – settings, dosages, duration, and clinical outcomes: a narrative review

**DOI:** 10.1097/MS9.0000000000002112

**Published:** 2024-04-29

**Authors:** Athanasios Athanasiou, Marinos Charalambous, Theodora Anastasiou, Konstantina Aggeli, Elpidoforos S. Soteriades

**Affiliations:** a Department of Cardiothoracic Surgery, Nicosia General Hospital; b Department of Basic and Clinical Sciences, University of Nicosia Medical School; c Department of Medicine, University of Cyprus; d Healthcare Management Program, School of Economics and Management, Open University of Cyprus, Nicosia, Cyprus; e Department of Environmental Health, Environmental and Occupational Medicine and Epidemiology, Harvard T.H. Chan School of Public Health, Boston, Massachusetts, USA; f Department of Cardiology, ‘Hippocrates’ General Hospital, National and Kapodistrian University of Athens, Athens, Greece

**Keywords:** ascorbic acid, cardiac surgery, postoperative, preoperative, vitamin C

## Abstract

Vitamin C or ascorbic acid is a water-soluble vitamin capable of directly donating electrons to reactive oxygen species, attenuating electrical remodeling, and cardiac dysfunction in patients undergoing cardiac surgery (CS), considered one of the most effective defenses against free radicals in the blood, thus being one of the first antioxidants consumed during oxidative stress. The aim of this review is to assess the effects of perioperative administration of vitamin C in CS patients. A comprehensive literature search was conducted in order to identify prospective cohort studies and/or randomized controlled trials reporting on the perioperative effects of vitamin C among adult patients undergoing CS. Studies published between January 1980 to December 2022 were included in our search, resulting in a total of 31 articles that met all our inclusion criteria. There seems to be a beneficial effect of vitamin C supplementation in arrhythmias such as in postoperative atrial fibrillation, reduction of ICU length of stay, and hospital length of stay, reduction in postoperative ventilation time, in inotropic demand, and in postoperative fatigue. Vitamin C can act as a scavenger of free radicals to decrease the peroxidation of the lipids present in the cell membrane, and to protect the myocardium postoperatively from ischemia/reperfusion injury, thus attenuating oxidative stress and inflammation. It represents a readily available and cost-effective strategy that could improve the outcome of patients undergoing CS, by reducing the risk of serious cardiovascular adverse events, both perioperatively and postoperatively.

## Introduction

Highlights

- In the current review (31 articles), we summarize the effects of preoperative and postoperative administration of vitamin C among adult cardiac surgery (CS) patients.
- There is a reduction of plasma concentration of vitamin C after a CS and needs to be substituted.
- Vitamin C attenuates oxidative stress and inflammation, thus protecting myocardium from ischemia/reperfusion injury.
- There is evidence for a beneficial effect of vitamin C supplementation in arrhythmias such as in postoperative atrial fibrillation, reduction of ICU length of stay, and hospital length of stay, reduction in postoperative ventilation time, in inotropic demand, and in postoperative fatigue.
- Substitution of vitamin C postoperatively, is a strategy that could improve the outcome of patients undergoing CS.

Vitamin C or ascorbic acid (AA) is a water-soluble vitamin that has stimulated widespread scientific interest, since its discovery, thanks to its various functions^1^ within the human body, impacting nervous, respiratory, renal, and cardiovascular systems, as well as coagulation, hemoglobin formation, inflammation, and immunomodulation (Table 1)^14,38,39^. Vitamin C is a lactone, comprising 6 carbon, 8 hydrogen, and 6 oxygen molecules linked with double and single covalent bonds (C6H8O6)^40^ and acts as an electric donor (or reductant) in over 60 enzymatic pathways^41^. Different studies reveal its contribution to the reduction of oxidative stress and organ dysfunction making it widely known as one of the most significant antioxidants^12^. Its chemical structure can explain its antioxidant effects and its ability to control the intracellular redox equilibrium^42^, protecting cells and organs from damage to macromolecules^43^. It leads to the formation of semidehydro-AA, which is a stable acid that is further metabolized and discharged^5^. The ascorbate anion (ASC), through its interaction with glutathione (GSH)^42,44–47^, can directly donate electrons to reactive oxygen species (ROS), such as O=O singlet oxygen, superoxide anions, hydroxyl radicals, and tocopheroxyl radicals^48^. This spontaneous electron provision to free radicals eliminates them efficiently^47^ and therefore relieves the oxidized cellular environment caused by aerobic metabolism. Consequently, vitamin C attenuates electrical remodeling and this is crucial, as structural and electrical modifications that are generated by oxidative stress are substrates for cardiac dysfunction^49^.

**Table 1:** Summary of vitamin C influence on different systems

System	Effects of vitamin C
Cardiovascular system	Attenuates myocardial damage and improves myocardial stunning^2^. Restores vascular responsiveness to vasoconstrictors thus reducing vasopressor demand^3^. Contributes to the biosynthesis of noradrenaline, cortisol, angiotensin, and hormones that are vital for maintaining adequate vascular tone for the perfusion of various organs^4^. Reduces the rate of AF or other supraventricular and ventricular arrhythmias^5,6^. Improves endothelial function^7^. Reduces arterial stiffness^8^. Stimulates endothelial proliferation, inhibiting apoptosis, and spares endothelial cell-derived nitric oxide to help regulate blood flow. Maintains vascular responsiveness and integrity. Promotes the differentiation of embryonic and pluripotent stem cells into cardiac myocytes^9^. Improves micro-perfusion^10,11^. Improves ventricular function^12^. Reduces both SBP and DBP^13^. Lowers blood cholesterol levels^14^, inhibits oxidation of LDL-protein, thereby reducing atherosclerosis and preventing CAD^15^.
Coagulation system	Prevents microthrombus formation^10^. Restores platelet function, decreases capillary plugging, reverses platelet-endothelial adhesion^10^. Attenuates a sepsis-induced drop of thrombocytes^10^. Facilitates enteral uptake of non-heme iron, and reduction of folic acid^1^.
Eye health	Slows down the progression of advanced age-related macular degeneration and loss of visual acuity^16^. Protects against cataract^17^.
Immune system	Bacteriostatic activity (inhibited bacterial replication)^11,18^. Supports endothelial barrier function and promotes antioxidant scavenging^18^. Enhances the proliferation and differentiation of B and T-cells^18^. Promotes antioxidant scavenging activity of the skin^19^. Shortens time for wound healing^18^ (by synthesis of collagen)^1^.
Gastrointestinal system	Attenuates drug toxicity (e.g. chemotherapy drugs), decreases inflammatory reaction^20^. Reduces the expression of apoptosis-related genes^21^. Protects against gastric cancers^22^. Promotes iron absorption in the small intestine^22^.
Musculoskeletal system	Inhibits osteoclast activity and stimulates osteoblast maturation^23^. Decreases postoperative pain and prevents complex regional pain syndrome (CRPS) following orthopedic procedures^24^. Reduce the feeling of fatigue in patients with cancer or inflammatory diseases^25^.
Nervous system	Elevated levels protect neurons from oxidative damage^2^. Reduces the infarct volume after ischemia^26,27^. Contributes to the myelination of the neurons^28^. Acts as a cofactor in neurotransmitter synthesis^29^.
Renal system	Reduces fluid demand and increases urine production^30,31^. Protects kidney function and renal arterial reactivity against IRI^32^. Decreases resistance index of the renal artery^32^.
Respiratory system	Reduces intubation time^33^. Decreases risk of pneumonia and alveolar inflammation^34^. Participates to the treatment of acute respiratory infections and to the mitigation of inflammation in critical COVID-19 patients^35^.
Reproductive system	Protects spermatogenesis, plays a major role in semen integrity and fertility, and prevents sperm agglutination^36^. Plays key role in the synthesis of testosterone^36^. Minimizes alterations in the male reproductive system caused by hyperglycemia^37^.

In the human body, vitamin C cannot be synthesized^50^, so it is provided entirely through the diet^51^ and is mainly acquired by the consumption of fruits (especially citrus), vegetables, and supplements^52^. Its molecular weight is 176.12 gr/mol (1 mg=5.7 μmol)^53^ and for healthy adults, a regular diet should include 40 mg of vitamin C per day [currently established recommended dietary allowance (RDA)]^54^. Current evidence suggests that low levels of vitamin C correlate with inflammation [C-reactive protein (CRP)], suggesting for high vitamin C consumption during oxidative stress. Low levels of vitamin C may be a risk marker of organ dysfunction (Table 2)^26,48,49,65^ as low levels are identified in patients with sepsis, hemorrhage, multiple organ failure, stroke, traumatic brain injury, and in patients undergoing cardiac surgery (CS)^66^ due to rapid leukocyte turnover and higher cellular consumption. This is interpreted by the fact that intracellular ascorbate concentrations in granulocytes and mononuclear leukocytes are 25 and 80 times higher than in plasma, respectively^1,47,48,67,68^. The physiologic levels of this micronutrient may be restored by intravenous vitamin C treatment, and/or high oral supplementation. Studies propose that people undergoing surgery require more than 500 mg of vitamin C per day, to prevent associated complications, while those who are under a lot of stress require even higher doses^69^.

**Table 2:** Consequences of vitamin C deficiency on different systems.

System	Consequences of vitamin C deficiency
Cardiovascular system	Increased risk of CHD^15,55,56^. Increased risk of acute myocardial infarction^15^. Decreased synthesis of hormones e.g. norepinephrine and epinephrine^57^. Increased progression of atherosclerosis^58^.
Coagulation system	Anemia, bleeding gums, capillary hemorrhage^14^ (petechial bleedings in skin, gingiva and sub-periosteally)^59^. Endothelial dysfunction^60^.
Immune system	Impaired immunity and higher susceptibility to infections^18^. Higher C-reactive protein levels^18^. T lymphocyte function impairment in cell-mediated cytotoxicity^61^.
Eye health	Hemorrhagic ocular pathologies.
Gastrointestinal system – Metabolism	Hypercholesterolemia, cholesterol gall stones formation^14^. Mild hypertriglyceridemia, depressed mobilization of nonesterified fatty acids^62^. Centrilobular fatty degeneration, moderate hyperplasia of the bile ducts and discrete signs of liver fibroplasia^62^. Development of alcoholic cirrhosis^63^. Increased risk of diabetes mellitus^60^.
Musculoskeletal system	Osteolysis, osteonecrosis, bone loss and pathological fractures^23^. Development of osteoporosis^58^. Poor collagen synthesis and other extracellular matrix proteins (e.g. glycosaminoglycans)^14,57^.
Nervous system	Unspecific mood disorders and depression^59^. Peripheral neuropathy^57^. Reduced cognitive performance in children^59^. Severe cerebellar hemorrhages^57^.
Respiratory system	More susceptible to severe respiratory infections such as pneumonia^35,58^.
Reproductive system	Reduction in reproductive performance^36^.
Skin health	Weakness of collagenous structures^18^. Skin fragility, bleeding gums, corkscrew hairs, impaired wound healing^19,64^.

Even though, vitamin C is considered as a first-line supplement^22,70–72^, its clinical use and therapeutic effectiveness for patient recovery and improvement of long-term clinical outcomes in CS patients is still under investigation^73–77^. Therefore, in the current review, we sought to summarize the effects of preoperative and postoperative administration of vitamin C among adult CS patients. Abbreviations and acronyms used throughout this manuscript are explained in Table 3.

**Table 3:** Abbreviations and acronyms used in the review

AA	=	Ascorbic acid	LDH	=	Lactate dehydrogenase
AAD	=	Antiarrhythmic drug	LOS	=	Length of stay
ABG	=	Arterial blood gas	LVET	=	Left ventricular ejection fraction
Ach	=	Acetylcholine	m	=	Months
AE	=	Adverse event	MDA	=	Malondialdehyde
AF	=	Atrial fibrillation	MFIS	=	Modified fatigue impact scale
AKI	=	Acute kidney injury	MI	=	Myocardial infraction
ASA	=	American Society of Anesthesiologists	NAC	=	N-Acetylcysteine
ASC	=	Ascorbate anion	NADPH	=	Nicotinamide adenine dinucleotide phosphate
b.i.d	=	Twice daily	ND	=	Non-defined
BP	=	Blood pressure	NF	=	Nuclear factor
CABG	=	Coronary artery bypass graft surgery	NO	=	Nitric oxide
CBC	=	Complete blood count	NKF	=	National kidney foundation
CHD	=	Coronary heart disease	NS	=	Non-significant
CHF	=	Congestive heart failure	NSAID	=	Nonsteroidal anti-inflammatory drugs
CI	=	Confidence interval	NYHA	=	New York Heart Association
CK-MB	=	Creatine kinase MB	o.d.	=	Once daily
COPD	=	Chronic obstructive pulmonary disease	oLAb	=	Antibodies against oxidized LDL
CPB	=	Cardiopulmonary bypass	OM	=	Outcome measures
CPK	=	Creatine phosphokinase	OP	=	Operation
Cr	=	Creatinine	OR	=	Odds ratio
CRP	=	C-reactive protein	PAD	=	Peripheral artery disease
CS	=	Cardiac surgery	PLT	=	Platelets
d	=	Days	p.o.	=	Oral administration
DB	=	Double blinding	POAF	=	Postoperative atrial fibrillation
DBP	=	Diastolic blood pressure	POD	=	Postoperative day
DM	=	Diabetes mellitus	Postop	=	Postoperative/Postoperatively
EC	=	Exclusion criteria	Preop	=	Preoperative/Preoperatively
ECC	=	Extracorporeal circulation	Pros	=	Prospective
ECG	=	Electrocardiography	PUFAs	=	Polyunsaturated fatty acids
EF	=	Ejection fraction	PVRI	=	Pulmonary vascular resistance index
eGFR	=	Estimated glomerular filtration rate	r	=	Correlation coefficient
Euro SCORE	=	European System for Cardiac Operative Risk Evaluation	qds	=	Four times daily
ESR	=	Erythrocyte sedimentation rate	RBC	=	Red blood cells
GSE	=	Grape seed extract	RCT	=	Randomized controlled trial
GSH	=	Glutathione	RDA	=	Recommended dietary allowances
H	=	Hours	ROS	=	Reactive oxygen species
Hb	=	Hemoglobin level	SBP	=	Systolic blood pressure
HDL	=	High density lipoprotein	SD	=	Standard deviation
HF	=	Heart failure	SIRS	=	Systemic inflammatory response syndrome
HLOS	=	Hospital length of stay	SOD	=	Superoxide dismutase
HR	=	Heart rate	SPECT	=	Single photon emission computed tomography
IC	=	Inclusion criteria	SR	=	Sinus rhythm
ICULOS	=	Intensive care unit length of stay	SS	=	Statistically significant
IL6/8	=	Interleukin 6/8	StR	=	Study regimen
IRI	=	Ischemia reperfusion injury	TAC	=	Total antioxidant capacity
IV	=	Intravenous	TB	=	Triple blinding
K/DOQI	=	Kidney disease outcomes Quality initiative	Vit	=	Vitamin
LAH	=	Left atrial hypertrophy	WBC	=	White blood cells

## Materials and methods

A comprehensive literature search was conducted in four major electronic databases (Medline/PubMed, Cochrane Library, Embase/Elsevier, and Google Scholar) in order to identify prospective cohort studies and/or randomized controlled trials (RCTs) reporting on the preoperative and postoperative administration of vitamin C among CS patients. The search covered the period between January 1980 to December 2022.

Predefined search terms included ‘vitamin C’, ‘ascorbic acid’, ‘ascorbate’ and ‘cardiac surgery’, ‘cardiothoracic surgery’, ‘heart surgery’, ‘cardiopulmonary bypass’, ‘CPB’, ‘extracorporeal circulation’, ‘ECC’, ‘coronary artery bypass grafting’, ‘CABG’, ‘CAB’, ‘valve surgery’, and ‘valvular surgery’. There was no language limitation. Studies that were included in the analysis met the following criteria: (i) prospective cohort studies and/or RCTs, (ii) adult patients (>18 years old) undergoing CS, (iii) comparison of antioxidant vitamin C with a control or placebo group, (iv) studies that reported data on the preoperative and/or postoperative administration of vitamin C (duration and dosage), and (v) studies that reported information on the incidence of postoperative complications following administration of vitamin C in adult CS patients.

Studies were excluded if they reported on nonadult patient populations (<18 years old). Review articles focusing on the topic were excluded, however, the reference list of all review articles identified, was searched in order to include any additional original articles that met the inclusion criteria and were not identified through the extensive databases’ search.

## Results

In brief, a total of 132 published articles were identified through the initial literature search from the four major electronic databases. Following the abstract review, we excluded 101 manuscripts that did not include clinical events based on the principal inclusion and exclusion criteria. The 31 remaining articles were examined in full-text and included in the present narrative review since they met our inclusion criteria. All enrolled studies reported on vitamin C administration regimens (solely or amongst with other antioxidants) as well as on the preoperative and postoperative effects among CS patients: namely the clinical setting, dosages, duration, potential complications, and/or the primary or secondary clinical outcomes. A detailed list of the enrolled studies with their characteristics and outcome measures (OMs) is presented in Table 4.

**Table 4:** Characteristics of studies included in the review

Author, year of publication, country, type of study, OP type, study groups, mean age (Years), male sex (♂)	Inclusion criteria (IC), exclusion criteria (EC), study regimen (StR), outcome measures (OM)	Results comments
Alshafy^78^ 2017 Egypt RCT OP: CABG | CPB: ND Group 1 (Vit C): 50 Group 2 (Control): 50 Total: 100 Mean age: Group 1: 54.58±6.00 | Group 2: 55.88±5.37 ♂: Group 1: 54% | Group 2: 60%	IC: ischemic heart disease underwent elective CABG, EF >30%, one to four grafts, without other cardiac disease, without ischemic mitral regurgitation. EC: underwent CABG before, emergency CABG, patients with EF <30%, contraindications to b-blockers or AA. StR: Group 1: bisoprolol 5 mg o.d. & VitC 2 g daily qds for at least 3 days preop. Group 2: bisoprolol 5 mg o.d. for at least 3 days preop. Route ND. OM: POAF, ICULOS, Ventilation time, Inotropic demand	POAF (1st day and after 48h): Vit C: 5/50 (10%) | Control: 14/50 (28%), *P*=0.022 POAF (after 1-month): Vit C: 2/50 (4%) | Control: 9/50 (18%), *P*=0.025 AF at discharge: SS, *P*=0.025 ICULOS (d): Vit C: 2.78±1.33 | Control: 3.58±0.73, *P*=0.035 Ventilation time >24 h: VitC: 14/50 (28%) | Control: 17/50 (34%), *P*=0.048 Inotropic demand 24 h postop: Vit C: 22/50 (44%) | Control: 32/50 (54%), *P*=0.045 Complications (renal, neurological, bleeding): NS.
Angdin^79^ 2003 Sweden RCT DB OP: CS | CPB: 100% Group 1 (Treatment): 12 Group 2 (Placebo): 10 Group 3 (Controls): 16 Total: 38 Mean age: Group 1: 72 |Group 2: 66 | Group 3: 66 ♂: 28/38	IC: adults undergoing elective CS, PVRI before surgery decreased by >5% after infusion of Ach. EC: DM, treated with acetylcysteine, allopurinol, and vitamins C and E. StR: Group 1: 900 mg VitE daily for 10 to 14 days before surgery plus 2 g Vit C and 300 mg allopurinol the evening before surgery plus 300 mg VitE, 2 g VitC, and 600 mg allopurinol on the day of surgery. During surgery infusion of Ach until 2 h after surgery. Group 2: only Ach. Group 3: no Ach (but saline). OM: Pulmonary reactivity to an infusion of Ach - Pulmonary vascular resistance index (PVRI), Markers of oxidative stress (Lipid peroxides, total antioxidant activity, diene conjugates)	Reduction in PVRI induced by ACH: Ach decreased mean pulmonary artery pressure in the treatment group only. Lipid peroxides (pmol/mg protein): Treatment: Preop: 5.16±1.24 | Postop: 16.69±2.15, *P*<0.0001 Placebo: Preop: 7.38±1.94 | Postop: 17.50±1.90, *P*<0.0001 Total antioxidant activity (%): Treatment: Preop: 37.6±0.7 | Postop: 31.5±1.6, *P* = 0.0007 Placebo: Preop: 36.9±0.9 | Postop: 30.1±0.9, *P*=0.0002. In cardiac patients receiving antioxidants the endothelium-dependent vasodilation was better maintained after surgery
Antonic^80^ 2016 Slovenia RCT OP: CABG | CPB: 100% AA: 52 Control (standard of care): 53 Total: 105 Mean age: AA: 64.60±7.29 |Control: 65.02±9.12 ♂: AA: 80.8) |Control: 75.5%	IC: elective on-pump CABG surgery. EC: emergency operations, concomitant valve or other surgery, history of AF, permanent pacemaker, hyperoxaluria or history of nephrolithiasis, off-pump surgery. StR: Preop: 2×2 g: 24 and 2 h before surgery IV Postop: 2×1 g/day for 5 days IV b-blockers: AA: 75.5 preop | Control: 78.8 preop Amiodarone: AA: 1.9 preop |Control: 1.9 pre Statins: AA: 88.7 preop |Control: 92.3 preop OM: POAF, ICULOS, HLOS	POAF: Vit C: 13.5% |Control: 18.9%, *P*=0.314, NS Mean duration of AF (h): Vit C: 11.2±9.7 | Control: 10.4±13.1, *P*=0.649 Time of occurrence of AF (POD): VitC: 3.71±1.89 | Control: 2.91±1.58, *P*=0.342 ICULOS (d): VitC: 1.50±1.06| Control: 1.30±0.69, *P*=0.437 HLOS (d): VitC: 9.37±.57| Control: 8.77±1.40, *P*=0.561. The results of this study do not support the effectiveness of AA supplementation in reducing the incidence of POAF in elective on-pump CABG patients.
Antonic^81^ 2017 Slovenia RCT OP: CABG | CPB: 100% Vitamin C: 50 Control: 50 Total: 100 Mean age: 65.02 ± 9.12 ♂: 78.1%	IC: scheduled elective on-pump CABG EC: severe preexistent acute or chronic kidney disease (NKF-K/DOQI) stages 4 & 5, eGFR <30 ml/min/1.73 m^2^, emergent surgery, critical preop state (Euro SCORE II), adjuvant valve surgery, exposure to contrast medium within 1-week preop, use of vitamin C within 1-month preop, off-pump surgery StR: Preop: 2×2 g 24 and 2 h before surgery IV Postop: 2×1 g/day for 5 days IV OM: POAF, ICULOS, HLOS, AEs, Postop AKI for 5 POD	Postop AKI: VitC: 16% |Control: 14%, *P*=0.779 Postop serum Creat (µmol/l): VitC: 83 | Control: 83, *P*=0.434 ICULOS (d): VitC: 1.42±1.01 | Control: 1.38±0.75, *P*=0.902 HLOS (d): VitC: 9.32±2.52 | Control: 8.80±1.58, *P*=0.582. NS protective effect of AA on the incidence of postop AKI. Limitations: sample size was relatively small with only 100 patients included, short-term, (5 postop days), single-center trial.
Bjordahl^82^ 2012 USA RCT TB OP: CABG | CPB: 170/185 VitC: 89 Control (placebo): 96 Total: 185 Mean age: Vit C: 63±12.4 |Control: 63±12.4 ♂: VitC: 68.5% |Control: 65.6%	IC: >18 years old, undergoing nonemergency CABG. EC: current AF, pacemaker, allergy to AA or any component of the trial supplement, pregnancy, emergency surgery, life expectancy <1-month, inability to take the trial AA supplement orally or by enteral tube. StR: Preop: 1×2 g PO night before surgery Postop: 2×1 g/day PO or enteral tube for 5 postop days b-blockers: AA: 82 pre | Control: 78.1 pre OM: POAF, ICULOS, HLOS, AEs, Ventilator days, Vasopressor use, Death	POAF: VitC: 27/89 –30.3% |Control: 29/96–30.2% *P*=0 .985 Ventilator time (d): VitC: 1.2±0.8 |Control: 1.4±1, *P*=0.032 ICULOS (d): VitC: 3.7±2.2|Control: 4.3±2.9, *P*=0.155 HLOS (d): VitC: 10.4±4 |Control: 11.7±7.1, *P*=0.124 Mortality: VitC: 2/89–2.2% |Control: 4/94–4.2%, *P*=0.836 Supplementation of AA in addition to routine postop care does not reduce AF after CABG
Carnes^83^ 2001 USA Pilot retrospective OP: CABG | CPB: ND VitC: 43 Control: 43 Total 86 Mean age: VitC: 61.6±12.2 |Control: 61.8±11.2 ♂: VitC: 83.7% |Control: 83.7%	IC: scheduled for primary CABG. EC: abnormal renal dysfunction not in sinus rhythm (preop AF). StR: Preop: 1×2 g VitC PO the night before surgery Postop: 2×500 mg PO for 5 days after surgery b-blockers: AA: 86 pre, 84 post | Control: 76 pre, 70 post. OM: POAF or atrial flutter for 5 POD.	POAF: VitC: 7/43–16.3%| Control: 17/43– 34.9%, *P*=0.048 B-blocker use has the most protective effect (84% risk reduction, *P*=0.007) and suggests that AA usage alone (66% risk reduction, *P*=0.09) would also have a clear beneficial effect. Weakness: not all risk factors were matched between groups as the incidences of hypertension, diabetes and prior AF were higher in the control group. Patients not blinded to treatment. No consistency in length of postop treatment with AA
Castillio^49^ 2010 Chile RCT DB OP: CS | CPB: 100% VitC: 48 Control: 47 N=95 Mean age: 59 years ♂: 68/95	IC: elective on-pump surgery, sinus rhythm. EC: AF, congenital or previous heart surgery, cirrhosis, chronic renal failure (Cr>150lM), inflammatory or rheumatic diseases, corticosteroid users, use of anti-inflammatory or antioxidant actions, use fish oil supplements 3 months previously. StR: N-3 PUFAs 2 g/day for 7 days before, VitC 1 g/day & Vit E 400 IU/day from 2 days before surgery all until discharge. OM: POAF, GSH ⁄ GSSG, MDA, Protein carbonylation, Leukocyte count, high sensitivity (hs) -CRP, Nuclear factor (NF) - kappa B activation	POAF: VitC: 11/48–22.9% | Control: 15/47 – 31.9%, *P*=0.32 HLOS (d): VitC: 8.15±0.16 |Control: 9.20±0.18, *P*=0.02 Lipid peroxidation (MDA) and Protein carbonylation were 27.5% and 24% lower in supplemented patients (*P*<0.01). Leukocyte count and serum hs-CRP levels were markedly lower throughout the protocol in supplemented patients (*P*<0.01). The combined n-3 PUFAs-antioxidant vitamin protocol therapy reduced the oxidative stress and inflammation biomarkers, in patients undergoing on-pump CS
Colby^84^ 2011 USA RCT DB OP: CS | CPB: 50% VitC: 13 Control (placebo): 11 Total: 24 Mean age: 65.5 ± 8.8 ♂: 79%	IC: >18 years old, undergoing CABG±valve surgery. EC: pregnant, history of renal calculi, hypersensitivity to AA StR: Preop: 1×2 g PO night before Postop: 2×0.5 g/day PO the day of surgery to postop 4th day b-blockers: AA: 54 pre, 85 post |Control: 55 pre, 100 post Amiodarone: AA: 36 post |Control: 27 post Statins: AA: 77 pre, 77 post | Control: 73 pre, 91 post OM: POAF, ICULOS, HLOS, AEs, Inflammatory mediators (CRP, WBC, Fibrinogen).	POAF: VitC: 31% |Control: 45%, *P*=0.498, NS ICULOS (h): VitC: 106.4±249.9|Control: 48.9±43.2, *P*=0.461 HLOS (days): VitC: 14.6±17.6 |Control: 10.2±4.9, *P*=0.429 [CRP] (mg/dl): PO4 Vit C: 14.5±7.8 | Control: 20.4±9.7 On-pump: 16.8±5.9| Off-pump: 17.8±11.5 WBC count (1000 cells/mm): POD4 Vit C: 9.4±3.1 | Control: 9.7±3.0 On-pump: 9.9±2.8| Off-pump: 8.8±3.3 Fibrinogen (mg/dl): POD4 Vit C: 673.9±190.1| Control: 684.6±239.7 On-pump: 680.4±204.6| Off-pump: 667.4±183.4
Das^85^ 2016 India RCT D OP: CS | CPB: 100% VitC: 35 Control (placebo): 35 Total: 70 Mean age: VitC: 48.14±6.46|Control: 45.23±11.47 ♂: ND	IC: free of any endocrine disease, aged 25–60 years old, NYHA status <IV, scheduled for elective CS. EC: parsonnet score >10, use of steroids, DM, alcohol abuse, smokers, pregnancy, epilepsy, hematological disease, impaired hepatic or renal function, hypersensitivity to any of the study drugs, requiring cross clamp time >2 h. StR: Preop: 2×0.5 g VitC po for 7 days prior to surgery till morning of surgery. OM: Cortisol level	Cortisol level 1st postinduction h: VitC: 69.51±7.65| Control: 27.74±4.72, *P*<0.05 Vasopressor demand: VitC: 2.83±1.27 | Control: 3.40±1.09, *P*=0.047. Time of extubation, ICULOS, incidence of arrhythmias was similar in both the groups. Adrenal suppression by etomidate was statistically significantly less in Vit c as compared to control group which is reflected by a higher level of cortisol in patients who received VitC
Dehghani^86^ 2014 Iran RCT OP: CABG | CPB: 100% VitC: 50 Control (no vitC): 50 Total: 100 Mean age: 61.31±6.42 ♂: 74%	IC: >50 years old, without history of CABG surgery, taking b-blocker, undergoing elective isolated on-pump CABG. EC: arrhythmia: using AADs or digoxin, severe chronic HF, LV EF<30%, pacemaker, renal (Cr>1.5) or hepatic failure, COPD, no occurrence of intra- or post-operative cardiopulmonary arrest, any degree of cardiac blockade, bradycardia <50 bpm. StR: Preop: 1×2 g Vit PO Postop: 2×0.5 g (500 mg) /day for 5 days PO B -blockers: all patients 1 wk before and continued postop Statins: AA: 18 pre, 14 post | Control: 20 pre, 16 post OM: POAF, ICULOS, HLOS, AEs	POAF: VitC: 4/50–8% | Control: 16/50–32%, *P*=0.003 Length of POAF (d): VitC: 2±0.816 | Control: 2.69±1.195, *P*=0.295 Time needed for converting AF to SR (h): VitC: 3.37±1.03| Control: 14±28.95, *P*=0.395 ICULOS (d): VitC 1.79±0.313 |Ctrl 2.10±0.61, *P*=0.002 ICULOS (d): AF: 1.80±0.30 |no AF: 2.56±0.69, *P*=0.001 HLOS (d): VitC 5.32±0.59 | Ctrl 5.74±1.3, *P*=0.041 HLOS (d): AF: 5.25±0.50 |no AF: 6.65±1.70, *P*=0.001 Ventilation time (h): VitC: 13.38±2.01 | Control: 15.41±14.35, *P*=0.325
Dingchao^43^ 1994 China RCT OP: CABG | CPB: 100% VitC: 45 Control: 40 Total: 85 Mean age: ND ♂: ND	IC: ND EC: ND StR: 2×125 mg/kg: 30 min before surgery and at the end of CPB (after aortic declamping) IV. Totally: 250 mg/kg OM: MDA, CPK, CPK-MB, LDH	Changes in serum MDA, CPK, CPK-MB, LDH in group of VitC were lower (*P*<0.05) than in control during and after CPB. CPK: VitC: 45/45 (100%) |Control: 35/40 (87.30%), *P*<0.05 ICULOS (h): VitC: 25±4.7 |Control: 45±8.5, *P*<0.05 HLOS (d): VitC: 35±5.9 | Control: 35±5.9, *P*<0.05
Eslami^87^ 2007 Iran RCT - No blinding OP: CABG | CPB: 100% VitC: 50 Control (no AA): 50 Total: 100 Mean age: 60.19±7.14 ♂: 67%	IC: >50 years old, taking b-blocker at least 1-week preop (for a target HR 60–70 bpm), scheduled for elective CABG. EC: history of AF, class I or III AAD or digoxin, pacemaker, AV block, bradycardia (HR <50 bpm), hepatic disease, end stage renal disease, severe pulmonary disease (pneumonia or COPD), severe hepatic disease (cirrhosis or fulminant hepatitis). StR: Preop: 2 g the night before surgery PO Postop: 2×1 g for 5 days PO. All patients received b-blockers for at least 1-week preop and continued postop. OM: POAF for 4 POD, ICULOS, HLOS, AEs	POAF: VitC: 2/50–4%|Control: 13/50–26%, *P*=0.002, OR 0.119, 95% CI: 0.025–0.558 ICULOS (h): VitC 55.2+/-38.4 |Ctrl 62.4+/-35.5, NS HLOS (d): VitC 6.54 (6.5) (+/-3.24 (3.2) | Ctrl 7.08 (7.0)+/-3.45 HLOS (d): AF: 8.9±3.9 |no AF: 6.5±3.1, *P*=0.02 ICULOS (d): AF: 3.23±1.92|no AF: 2.33±1.45, *P*=0.09. Combination of AA and b-blockers may be more effective in reducing POAF than b-blockers alone
Gholami^25^ 2019 Iran RCT TB OP: CABG | CPB: 100% Intervention: 80 Control (placebo): 80 Total: 160 Mean age: VitC: 61.41±10.51 | Control: 63.93±9.97 ♂: VitC: 55% | Control: 47.5%	IC: ≥18 years old, no prior use of antioxidants, no use of fish oil over the past 3 months, lack of diseases with high inflammatory components. EC: hypersensitivity to n-3 PUFAs or Vit C, developing heart attack during or following the surgery. StR: Preop: n-3 PUFAs 2 g/bid & Vit C 1 g/bid the day before. Post: 2 g n-3 PUFAs (1 g/bid) and 1 g VitC (500 mg/bid) until the 5th postop day. OM: Fatigue - MFI-20 scale	Fatigue score: Intervention: 62.01±4.06 | Control: 67.92±4.95, *P*<0.0001. Changes in general fatigue score: VitC: 1.42±1.15 | Control: 2.66±2.50. Changes in physical fatigue score: VitC: 1.99 0.23 |Control: 2.26±1.53, *P*<0.0001. Changes in reduced active score: VitC: 1.83±0.14| Control: 1.83±0.14, *P*=0.01. Changes in mental fatigue score: VitC: 3.00±2.16| Control: 3.32±2.44, *P*=0.586, NS Combination of vitamin C and n-3 PUFAs effectively reduces postop fatigue among patients who undergo CABG surgery
Gunes^88^ 2012 Turkey Retrospective OP: CS | CPB: 100% Intervention: 34 Control: 25 Total: 59 Mean age: Intervention: 57.29±2.50 | Control: 55.08±2.87 ♂: 45/59	IC: ND EC: ND StR: Preop: VitC 500 mg and VitE 300 mg on the day of surgery IV Postop: VitC 500 mg/day and VitE 300 mg/day for 4 days IV All patients received 500 mg of methylprednisolone (in CPB). OM: CRP, WBC count, serum lipids (HDL, LDL, triglycerides)	Control group: CRP ↑ POD1 (*P*<0.001) CRP+r with triglycerides D0 (*P*=0.009) and POD1 (*P*=0.021) WBC ↓POD2 (*P*=0.008) WBC+r with glucose POD2 (*P*<0.005) Study group: CRP -r with HDL (*P*=0.049) on POD1 CRP +r with triglycerides on the POD2 (*P*=0.017) Blood Glucose ↑ POD1 (*P*=0.021) WBC +r with VitC and VitE on the POD4 (*P*=0.027) VitC and VitE seem to display some anti-inflammatory effect
Jouybar^89^ 2012 Iran RCT OP: CABG | CPB: 100% Vit C: 20 Control (placebo): 20 Total: 40 Mean age: VitC: 56.9±10.3 | Control: 61.83±10.45 ♂: VitC: 14/20 | Control: 12/20	IC: scheduled elective CABG, ASA score I or II EC: pregnancy, infection, malignant disease, history of hypersensitivity reaction, hematologic disease or abnormal WBC, DM, major trauma or major surgery <6 m preop, EF<35%, use of immunosuppressive drugs, uncontrolled BP, recent myocardial infarction <6 m, renal failure. StR: Preop: 2×3 g 12–18 h preop and during CPB initiation (after induction of anesthesia) OM: Inflammatory markers (IL6 and IL8)	IL6 and IL8 increased significantly after starting CPB. The pattern of changes at such levels was similar in both groups. AA has no effect on the reduction of inflammatory cytokines (IL6 and IL8) during CPB and did not improve kidney function. It causes no improvement in hemodynamics (*P*=0.37), blood gas variables (*P*=0.21), and the outcomes of patients undergoing CABG
Knodell^90^ 1981 USA RCT DB OP: CS | CBP: ND VitC: 111 Control (placebo): 104 Total: 215 Mean age: VitC: 53.6±8.8 | Control: 54.1±9.9 ♂: ND	IC: scheduled CABG EC: ND StR: Preop: 4×800 mg/day VitC PO for 2 days before surgery Postop: 4×800 mg/day VitC PO for 2 weeks postop started as soon as patient could take oral liquids OM: Posttransfusion hepatitis (liver enzymes)	Posttransfusion hepatitis: VitC: 6.7% | Control: 9.4%, *P*>0.50. significant elevations of plasma VitC in VitC treatment group, *P*<0.0005. Elevations of plasma VitC no influence on the hepatitis
Papoulidis^91^ 2011 Greece RCT DB OP: CABG | CPB: 100 VitC: 85 Control (placebo): 85 Total: 170 Mean age: VitC: 73.1±7.2 |Control: 71.3±6.8 ♂: VitC: 67% | Control: 74.1%	IC: >65 years old, NYHA Class III or IV HF, taking b-blocker, scheduled for elective isolated CABG. EC: age <65, preop AF, hyperoxaluria, permanent or temporary pacemaker, severe renal or hepatic failure, medication with class I and III AAD or digoxin, any degree of AV or bradycardia (HR <50 bpm), severe pulmonary disease (pneumonia or COPD). StR: Preop: 1×2 g 3 h prior to CPB IV Postop: 2×0.5 mg/day for 5 days IV All patients (100%) were on b-blockers both preop and postop. OM: POAF for 5 POD, HLOS, AEs	POAF: VitC: 38/85 (44.7%) | Ctrl: 52/85 (61.2%), *P*=0.041. Time needed for cardioversion in AF patients (h): VitC: 5±1.3 h | Control: 7.3±2.1, *P*=0.047 HLOS (d): AF: 9.5±2.8 | no AF: 6.7±1.9, *P*=0.034 HLOS (d): VitC: 7.9±2.2 |Control: 9.8±3.6, *P*=0.04 ICULOS (d): VitC: 1.6±0.9 | Control: 2.1±1.1, *P*=0.05 Combination of AA and b-blockers reduced the incidence of POAF
Sadeghpour^39^ 2015 Iran RCT OP: CS (CABG, Valve, Congenital) | CPB: 249/290 VitC: 113 Control (placebo): 177 Total: 290 Mean age: 55.78±13.72 ♂: VitC: 70.8% | Control: 62.7%	IC: ≥18 years old, undergoing CS, ASA II-III EC: severe cardiac, respiratory, or neurologic complications, emergency surgery, death on postop day 1, those who had not received adequate doses of drugs according to protocol. StR: Preop: 1×2 g immediately before surgery (at operating room) IV Postop: 1×1 g/day for 4 days postop PO OM: POAF, ICULOS, HLOS, AEs	POAF: VitC: 40/113 (35.3%) |Control: 99/177 (55.9%), *P* = 0.001 HLOS (d): VitC: 10.17±4.63|Control: 12±4.51, *P*=0.01 Intubation time (h): VitC: 11.83±3.91 |Control: 14.14±9.52, *P*=0.003 Drainage volume the 1st 24 h postop (cc): VitC: 262.21±190.91 |Control: 348.50±262.17, *P*=0.003. Postop complications: Significant difference, *P*=0.042. This trial suggests benefit from the routine early, prophylactic administration of VitC
Safaei^92^ 2017 Iran RCT OP: CABG |CPB: 100% Group 1 (no treatment): 29 Group 2: 29 Group 3: 29 Total: 8 Mean age: 57.07±1.05 ♂: 67/87	IC: elective isolated CABG EC: urgent patients, complicated high-risk patients, DM, another heart surgery beside CABG, ischemic time >120 min StR: Group 2: 4×100 mg Grape Seed Extract (GSE) 24 h before operation PO Group 3:25 mg/kg vitamin C during surgery iv OM: Total Antioxidant Capacity (TAC), Malondialdehyde (MDA), Superoxide Dismutase (SOD), Glutathione Peroxidase (Gpx)	ICULOS (h): VitC: 67.0±3.0| Control: 71.2±6.1, *P*=0.61 Tracheal intubation time (h): VitC: 15.1±1.0 | Control: 22.9±3.8, *P*=0.019 TAC: was higher (*P*<0.05) in both grape seed and VitC groups at T2 (ischemic period) and T3 times (Post-I/R period). MDA: in both GSE and VitC groups were always lower compared to control. GSE and VitC had antioxidative effects and reduced deleterious effects of CPB. No hospital mortality or AE occurred in study groups
Samadikhah^93^ 2014 Iran RCT DB OP: CS | CPB: ND VitC: 60 Control (placebo): 60 Total: 120 Mean age: VitC: 61.0±11.5 |Control: 60.5±11.3 ♂: VitC: 43/60 | Control: 39/60	IC: patients undergoing CABG EC: history of AF, valve disease, MI, EF <40%, LAH, heart valve disease, myocardial infarction StR: Preop: 1×2 g PO Postop: 1×1 g/day PO from 2nd until 5th POD Atorvastatin (for both groups): 40 mg PO until 5th postop day. b-blockers: AA: 75 pre | Control: 66 pre Statins: AA: 98.3 pre, 100 post | Control: 98.3 pre, 100 post OM: POAF	POAF: VitC: 6/60–10% | Control: 15/60–25%, *P*=0.03, OR=0.33, 95% CI=0.12–0.93 Combination prophylaxis against POAF with oral atorvastatin plus VitC is significantly more effective than single oral atorvastatin
Sisto^94^ 1995 Finland RCT OP: CABG | CPB: 100% Stable or Low risk: VitC: 20 | Control: 25 Unstable or High-risk: VitC: 17 | Control: 19 Total: 81 Mean age: Group 1: 54 ±8 | Group 2: 58±6 | Group 3: 55±8 | Group 4: 62±6 ♂: 56/81	IC: undergoing standard CABG. EC: women of premenopausal age, MI, use of study medicines during 30 days before CABG, renal (Cr >150 ~g/l) or hepatic (AST >40 IU/l) disease, allergy to study medicines. StR: Stable or low risk disease: Preop: 600 mg daily VitE for 4 weeks preop, 2 g daily VitC for 2 days before, 600 mg daily allopurinol for 2 days before PO Postop: 2 g daily VitC, 600 mg daily allopurinol for 1-day postop PO Unstable or high-risk: Preop: 600 mg daily VitE, 2 g daily VitC, 600 mg daily allopurinol for 2 days before PO Postop: 2 g daily VitC, 600 mg daily allopurinol for 1-day postop PO OM: Arrhythmias (AF) for 5 POD, CK-MB	Incidence of arrhythmias: High-risk patients: VitC: 12% | Control: 42%, *P*=0.05 Lower-risk patients: VitC: 25% | Control: 24%, *P*=ND ECG ischemic events: Low risk: VitC: 20% | Control: 52%, *P*=0.03 High-risk: VitC: 18% | Control: 58%, *P*=0.01 Postop MI: Low risk: VitC: 5% | Control: 8%, *P*=ND High-risk: VitC: 6% | Control: 32%, *P*=0.06 CK-MB release: lower in groups treated with antioxidants and allopurinol than in the controls. Vasopressor demand: control groups tended to require more dopamine than treatment groups (*P*=0.057). Combination therapy with allopurinol, vitamin C, and vitamin E appeared to inhibit ischemic ECG alterations and may protect against post-CABG arrhythmias
Stanger^95^ 2014 Australia RCT DB OP: CABG | CPB: ND Group 1 – Control: 20 Group 2 – Vitamins: 19 Group 3 – n-3 PUFAs: 19 Group 4 – Vitamins & n-3 PUFAs: 17 Total: 75 Mean age: 66±8 ♂: 68/75	IC: good pharmacological control, lipid profile, blood pressure values and glycemic control EC: ND StR: Group 1: Control (C) Group 2:500 mg VitC & 45IE VitE IV 30 m before reperfusion and 120 m after reperfusion Group 3:2×0.15 g fish oil/kg 42 h and 18 h before surgery and 50 ml 42 h after surgery IV Group 4: 2×0.15 g fish oil/kg 42 h and 18 h before surgery and 50 ml 42 h after surgery IV plus 500 mg vitC and 45IE vitE IV 30 m before reperfusion and 120 m after reperfusion OM: Serum oxidative stress biomarkers: Total peroxides, Endogenous peroxidase activity, Antibodies against oxidized LDL (oLAb)	POAF: Group 2: 5/19| Group3: 4/19| Group 4: 7/17 Vitamin supplementation was associated with a significant decrease in total peroxides both in Vit as well as in Vit & n-3 PUFAs, compared to C and n-3 PUFAs. Olab: significantly decreased only in C (*P*=0.002) at the POD1. Water-soluble and Lipid-soluble antioxidants: significantly decreased at the 1st and 2nd postop days in all subgroups but recovered at the 3rd postop day. Postop increase in oxidative stress was associated with the consumption of antioxidants and a simultaneous onset of AF. The administration of vitamins attenuates postop oxidative stress during CABG. In both subgroups that were supplemented with vitamins, total peroxides decreased
Rodrigo^96^ 2012 Chile RCT DB OP: CS | CPB: 100% VitC: 77 Control (Placebo): 75 Total: 152 Mean age >60: VitC: 66 |Control: 65 Mean age <60: VitC: 53 |Control: 52 ♂: VitC: 59/77 | Control: 61/75	IC: scheduled CS with extracorporeal circulation. EC: previous CS, chronic or paroxysmal AF, other preop arrhythmias, advanced pulmonary or hepatic disease, chronic renal failure (serum creatinine >2.0 mg/dl). StR: 2 g/d n-3 PUFAs 7 days before surgery and Vit C (1 g/day) and Vit E (400 IU/day) 2 days before and all continued until the discharge from hospital. OM: POAF until hospital discharge, Activity of antioxidant enzymes: Cu-Zn superoxide dismutase, catalase and GSH-Px activities, Plasma MDA	POAF: Patients <60 years old: VitC: 5/40–12.5% |Control: 12/40–30% Patients >60 years: VitC: 2/37–5.4% |Control: 10/35–2.8.5%, *P*=0.0076 Patients >60 years present higher levels of lipid peroxidation, lower enzymatic activity of GSH-Px and decreased total antioxidant plasma capacity. Patients with POAF showed higher plasma MDA levels postop and lower atrial GSH-px activity compared with those who did not present POAF (*P*<0.01). Antioxidant supplementation resulted in a more marked reduction of POAF in older patients. It could be suggested that the efficacy of this therapy improves with ageing
Rodrigo^97^ 2013 USA RCT DB OP: CS |CPB: 100% Intervention: 103 Control (Placebo): 100 Total: 203 Mean age: VitC: 61| Control: 58.5 ♂: VitC: 85| Control: 88	IC: ≥18 years old, scheduled for CABG or/and valve surgery, sinus rhythm. EC: history of any arrhythmia, previous MI, use of amiodarone or sotalol, severe CHF (NYHA III or IV), prosthetic valves, congenital valvular disease, left atrial diameter >50 mm, conditions associated with oxidative stress or inflammation such as chronic rheumatic or neoplastic diseases, liver insufficiency, severe chronic kidney disease (serum Cr >2.0 mg/dl), recent infections, use of NSAID, corticosteroids, antioxidant vitamins, or fish oil supplements 3 m preop. StR: N-3 PUFAs 2 g/day 7 days prior to surgery and 2 days before surgery added VitC 1 g/day and VitE 400 IU/day until discharge. OM: POAF, Biomarkers of oxidative stress and inflammation	POAF: Intervention: 10/13–9.7%| Control: 32/100–32%, *P*<0.001, RR: 0.28, 95% CI: 0.14– 0.56 Duration of POAF (h): VitC: 10.8±0.7 |Control: 11.2±1.5, *P*=0.06 ICULOS (d): VitC: 2.87±0.44| Control: 3.08±0.54 *P*=0.76 HLOS (d): VitC: 8.77±0.37|Control: 9.57±0.66 *P*<0.05. The activity of catalase, superoxide dismutase, and glutathione peroxidase in atrial tissue of the supplemented patients was 24.0%, 17.1%, and 19.7% higher than the respective placebo values (*P*<0.05). The atrial tissue of patients who developed AF showed NADPH oxidase p47-phox subunit protein and mRNA expression 38.4 and 35.7% higher, respectively, than patients in SR (*P*<0.05)
Westhuyzen^98^ 1997 Australia RCT DP OP: CABG | CPB: 100% Intervention: 38 Control (placebo): 38 Total=76 Mean age: 59.7±7.3 ♂: 82.9%	IC: elective CABG, aged 18–75, no smokers, gave informed consent >10 days preop. EC: receiving coumarin derivatives, vitamin supplements, corticosteroids, malabsorption syndrome, severe asthma, COPD, EF<40%, history of alcoholism, drug abuse, psychosis. StR: 750 IU VitE/day PO for 7 to 10 days prior to surgery and 1 g VitC PO 12 h before the operation. OM: CK-MB, Plasma a-tocopherol, Myocardial injury (ECG, SPECT)	Plasma [a-tocopherol] (mg/l): Preop: raised fourfold by supplementation. Intervention: 44.7% |Control: 11.4, *P*<0.001. Postop: fell by 70% in the supplemented group and to negligible levels in placebo group. CK-MB profiles: NS differences, *P*=0.71 ECG: Preop: The treatment group had significantly worse QRS scores than the placebo group at baseline (*P*=0.03). Postop: QRS scores were worse than preop scores in the combined group (*P*=0.001). SPECT: NS differences

There are only a handful of studies that give us an insight into the changes in plasma vitamin C concentrations following cardiac or more specifically coronary artery bypass grafting (CABG) surgery, as presented in Table 5. In these studies, the levels of plasma AA in mg/l or μmol/l were measured preoperatively as baseline measurements, and followed up for 6 h, up to 1-day, 2 days, or 14 days perioperatively and postoperatively. The pattern of plasma changes in all studies is surprisingly the same even after 2 weeks postoperatively. There is a reduction of plasma vitamin C following cardiopulmonary bypass (CPB), namely more than 50%, 6 h postoperatively, and the lower levels persist for up to 14 days^38,73,75,99–101^. Our search retrieved no study in the literature that examines vitamin C levels beyond the 2 weeks postoperatively in CS, neither has examined the effects of a longer-term supplementation in this group of patients. We hereby assume that since vitamin C is obtained entirely from diet, the vitamin C low plasma levels and in some cases even marginal deficiency^75^, will persist for weeks or even for longer periods if patients are undernourished or receive inadequate amounts of vitamin C supplementation. This can inevitably predispose this group of patients to the risks accompanying vitamin C low plasma levels or even deficiency, for example, progression of atherosclerosis^58^ with increased risk of coronary heart disease^15,55,56^ and even acute myocardial infarction (MI)^15^.

**Table 5:** Studies assessing changes in vitamin C following surgery

Study	Study design	*n*	Specimen type	Type of surgery	Follow-up time	Study results
Hill^38^ 2019	Pros	34	Plasma AA (VitC)	CABG w/ CPB	2 days	Reduced from 6.5 (3.5–11.5) mg/l to 2.8 (2.0–3.9) mg/l
Rodemeister^73^ 2014	Pros	29	Plasma AA (VitC)	Cardiac w/ CPB	6 days	Reduced from 61.5±18.6 μmol/l to 28±11.5 μmol/l
Valle-Giner^99^ 2007	Pros	27	Plasma AA (VitC)	Cardiac w/ CPB	6 h	Reduced from 43±3 μmol/l to 18±2 μmol/l
Lassnigg^100^ 2003	RCT	20 (placebo)	Plasma AA (VitC)	Cardiac w/ CPB	6 days	Reduced from 9.72±4.71 mg/l to 4.65±2.12 mg/l
Ballmer^75^ 1994	RCT	9 (control)	Plasma AA (VitC)	CABG w/ CPB	1, 14 days	Reduced from 36±19 μmol/l to 10.5 μmol/l (1-day) and 19.1 μmol/l (14 days)
Cavarocchi^101^ 1986	RCT	20 (control)	Plasma AA (VitC)	Cardiac w/ CPB	1-day	Reduced from 16±3 mg/l to 6.6±2.3 mg/l

### Characteristics and clinical settings of vitamin C studies

Vitamin C in the context of CS has been under investigation since the early beginning of the modern CS era in the late 60s. In our review, the literature extends from 1981 up to 2020. The geographic expansion of the studies includes almost all continents (North America, South America, Africa, Europe, Asia, and Australia). Notably there are far many studies from the Middle East, namely from Iran. The vast majority of the included studies were CABG operations with CPB. Depending on the various authors’ inclusion and exclusion criteria, clinical endpoints and scientific aspirations, sole valve/valves, or combined operations with CPB, and sparse off-pump CABG were met, all of which in notable smaller numbers and in fewer studies than those with sole CABG with CPB.

In the majority of studies, vitamin C was administered solely, whereas in some studies it was administered alongside with other antioxidants and/or anti-inflammatory or antiarrhythmic agents under various regimens. Amongst the standard of care for CS patients, n-3 polyunsaturated fatty acids (n-3 PUFAs) and/or vitamin E were used in various therapeutic schemes. Interestingly, the only two Scandinavian studies we have from Angdin *et al*.^79^ and Sisto *et al*.^94^ have implemented allopurinol in addition to vitamins C and E. The rationale of this combination is allopurinol’s ability to indirectly inhibit ROS generation^102^, thus acting as an antioxidant^103^, and theoretically conferring additive antioxidant capacity to the regimen.

Preoperatively, most of the studies used a single dose of 2 g of vitamin C with some variations among authors, especially among those administering combined therapies that followed no special pattern. Postoperatively, a smaller dosage of 1 g/day or less, was administered for some postoperative days, most frequently for 5 days. In general, vitamin C was administered in somewhat higher doses preoperatively and for shorter periods than postoperatively. There was only one author that administered solely vitamin C for up to 14 days postoperatively^90^. Rodrigo^96,97^ and Castillo *et al*.^49^ in their combined regimens (vitamins C and E and n-3 PUFAs) continued the regimen until hospital discharge. However, single dosages as high as 250 mg/kg have also been reported^43^. To our knowledge, there is no dose-finding study on CS patients yet.

### Cardio-protective effects of vitamin C

In patients with atherosclerosis, there is biochemical evidence suggesting increased oxidative stress, which results from an altered balance of endogenous pro-oxidants and antioxidants^104–106^. Vitamin C, the major water-soluble antioxidant in human plasma, has been shown to reverse endothelial dysfunction^107^ in patients with ischemic heart disease^108,109^ by restoring vascular responsiveness to vasoconstrictors^110^ while inhibiting nitric oxide (NO)-induced resistant vasodilation^10^. At the same time, it protects against oxidative stress-induced pathological vasoconstriction and protects against the loss of the endothelial barrier thereby preventing the depletion of endothelial nitric oxide (eNO)^1^. In vivo, ascorbate can directly inhibit platelet-endothelial adhesion in capillaries through its’ hemodynamic effects on capillary blood flow^10^. These functions include increasing the synthesis and deposition of type IV collagen in the basement membrane, stimulating endothelial proliferation, inhibiting apoptosis, scavenging free radicals, and sparing endothelial cell-derived nitric oxide, thus playing a beneficial role in endothelial growth, survival, function and maintenance of vascular responsiveness, and integrity^44^. Low-density lipoprotein (LDL) cholesterol in the blood can only be significantly atherogenic after oxidative modification, which allows it to be taken up by macrophages in the artery walls. Antioxidants such as vitamin C can protect LDL from oxidative modification and can help prevent coronary artery disease (CAD)^111^.

In the cardiovascular system, vitamin C is essential for the biosynthesis of noradrenaline, cortisol, angiotensin, and hormones that are vital for maintaining adequate vascular tone for the perfusion of various organs^4,112^. Ascorbate-dependent synthesis of vasopressors represents a plausible physiological mechanism whereby ascorbate could act as adjunctive therapy for severe sepsis and septic shock^112^. Observational studies have shown an inverse relationship between plasma vitamin C concentrations and peripheral arterial disease (PAD)^66^ or blood pressure^113,114^ as well as an inverse relationship between vitamin C intake and CAD mortality^115,116^, cerebrovascular events^117,118^, or PAD^119^.

The whole-body inflammatory response produced by CPB is a major pathogenetic cause for postoperative complications after cardiac procedures, and induces both oxidative stress and systemic inflammatory response^120–122^. The inflammatory response has been shown to produce a large number of ROS, cytokines, and other cytotoxic materials^89^. Re-exposure of tissues to ROS at the end of CPB produces the so-called ‘ischemia-reperfusion injury’ (I/R)^123^. These aggressive oxygen species can damage various cellular components and the damage can be continued by a chain reaction of peroxidation of PUFAs^124^. However, a multiple defense systems, including enzymes (e.g. superoxide dismutase), endogenous antioxidants (e.g. glutathione and serum proteins), and exogenous dietary antioxidants (e.g. vitamin C and E and carotenoids)^12^, protects the body from injuries caused by ROS^95,124^. Following reperfusion, the patient’s own natural antioxidant defense mechanisms prevent the release of free radicals. This implies the consumption of antioxidant nutrients, such as vitamins A, C, and E, selenium, glutamine, and zinc^12^. Depletion of these protective vitamins may potentially cause deleterious effects of ROS on organs’ functions after CPB. Based on its redox-potential and powerful antioxidant capacity, vitamin C, has been called ‘the most important antioxidant’, as it counters the influence of free radicals in the blood in the most effective way^125,126^, as well as it is the first antioxidant consumed during the oxidative stress, therefore sparing other endogenous antioxidants^125,126^. The finding of substantial vitamin C consumption suggests that a perioperative/postoperative vitamin C supplementation, in combination with other antioxidants, may moderate the whole-body inflammatory response and thereby reduce morbidity associated with CPB and CS^3,33,43,127,128^. Consequently, vitamin C is a promising ‘candidate’ for prophylactic and therapeutic interventions in clinical situations in which the balance between ROS and antioxidants is likely to be disrupted^75^, as many authors reported for postoperative atrial fibrillation (POAF)^5,6,87,91,94,97,129–132^.

Oxidative stress associated with heart failure (HF) leads to depletion of vitamin C^133–135^. Therefore, it has been proposed that vitamin C plays a role in HF therapy^136^ by restoring heart’s ventricular function^12^. There are many studies revealing its effects on the heart’s mechanical efficiency and contractility as assessed by LVEF (left ventricular ejection fraction) measurements^137–139^. Firstly, as shown in a meta-analysis conducted by Hemila *et al*^140^., vitamin C increased LVEF in both cardiac and noncardiac patients on average by 12 and 5%, respectively. Additionally, Shinke *et al*. observed that in people suffering from HF due to previous MI, administration of vitamin C improves the response of LV contractility and LV stroke work to adrenergic stimulation and thereby restores myocardial efficiency^141^. These results are also reinforced by the RCT conducted by Qin *et al*.^142^ who documented that vitamin C attenuated LV dilation and dysfunction in HF following MI through diminishing oxidative stress and reducing myocyte apoptosis. Moreover, Hornig’s *et al*.^143^ research demonstrated that long-term oral administration of vitamin C may improve endothelial dysfunction in congestive HF patients. Seemingly, alongside the conventional and newer therapeutic agents used for the treatment of HF^144,145^, vitamin C can have a beneficial role.

### Clinical outcomes

Taking into account all the aforesaid beneficial effects of vitamin C, we have focused on various relevant OMs. In most of the studies, the OMs were more or less similar and included arrhythmias (POAF or atrial flutter), ICU length of stay, hospital length of stay, ventilation time, inotropic demand, various adverse events (AEs), postop occurrence of acute kidney injury, postoperative fatigue, inflammatory mediators and blood markers [CRP, white blood cells, interleukin 6 & 8 (IL6 & IL8)], heart and serum biochemical markers [creatine phosphokinase (CPK), creatine phosphokinase – myocardial band (CPK-MB), lactate dehydrogenase (LDH), high density lipoprotein (HDL)], biomarkers of oxidative stress, lipid peroxides, and peroxidation as well as antioxidant capacity measures [Malondialdehyde (MDA), GSH/Glutathione disulfide (GSSG) ratio, diene conjugates, total antioxidant capacity (TAC), superoxide dismutase (SOD), glutathione peroxidase (Gpx), antibodies against oxidized LDL (oLAb)].

Though not consistent in all authors, there seems to be a beneficial effect of vitamin C supplementation in arrhythmias such as in POAF/flutter, reduction of ICULOS and HLOS, reduction in postoperative ventilation time, inotropic demand, and quite interestingly less postoperative fatigue. Gholami *et al*.^25^ found that immediately after CABG surgery, antioxidants with vitamin C can effectively improve patients’ general and physical fatigue as well as improve reduced activities and motivation to live. Sadeghpour *et al*. even reported less drainage volume in the first postoperative day (POD - 1) for the vitamin C group. An additive beneficial effect with regards to the reduction of postoperative arrhythmias was observed with simultaneous supplementation of vitamin C and b-blockers^83,87,91^, while Samadikhah *et al*.^93^ reported the same beneficial effect for the combination of atorvastatin plus vitamin C, than single oral atorvastatin.

While Dingchao *et al*.^43^ suggested that vitamin C can act as a scavenger of free radicals to decrease the peroxidation of the lipids present in the cell membrane and remove the radicals to effectively protect the myocardium I/R injury during and after open heart surgery, Jouybar *et al*.^89^ on the other hand, claimed that vitamin C has no effect on the reduction of inflammatory cytokines (IL6 and IL8) during CPB^89^. Nevertheless, the results from the Westhuyzen trial regarding the reduction of myocardial damage evaluated with ECG and single photon emission computed tomography (SPECT), in patients treated with vitamin C, were not proven to be statistically significant^98^.

Lastly, a number of combination studies that included vitamin C resulted in various promising findings^49,94–97^. Sisto *et al*., showed that a combination therapy with allopurinol, vitamin C, and vitamin E inhibited ischemic ECG alterations and protected against post-CABG arrhythmias. In the same study, the aforesaid therapy proved to be able to reduce postop MI in high-risk patients, while showed a trend for reduced vasopressor demand postoperatively as well as lower CK-MB release in groups treated with antioxidants and allopurinol compared to controls^94^. Stanger *et al*. administered a combination of vitamins C & E & n-3 PUFAs and examined serum oxidative stress biomarkers and oLAb. He found that water- and lipid-soluble antioxidants, significantly decreased at the 1st and 2nd postoperative days in all study subgroups but recovered at the 3rd postoperative day. The authors documented that vitamins C and E supplementation was associated with a significant decrease in total peroxides both in the vitamin as well as in the vitamin + n-3 PUFAs groups as compared to controls and n-3 PUFAs alone, thus concluded that the administration of vitamins C and E attenuates postop oxidative stress during CABG^95^. The same combination was examined by Rodrigo *et al*.^96^, who found that the antioxidant supplementation with n-3 PUFAs and vitamins C and E resulted in a more marked reduction of POAF in older patients. The same author in another study with the same combination of supplements showed that this safe, well-tolerated, and low-cost regimen favorably affected POAF, increased antioxidant potential, and attenuated oxidative stress and inflammation^97^.

### Adverse health effects

Vitamin C’s pharmacokinetics is complex and its plasma concentration depends on absorption, tissue transportation, volume distribution, cellular uptake, consumption (rate of utilization), and renal reabsorption and excretion^146^. The concentration dosage curve has a sigmoidal shape. Its steep portion ranges between 30 mg and 100 mg of daily vitamin C and 70 to 85 μmol/l are plasma concentrations plateau. Additional increases in plasma concentrations are minimal at dosages larger than 400 mg/day^147^. Overall, at maximum oral intake, plasma concentrations of vitamin C do not exceed 220 μmol/l. At plasma concentrations below 20 μmol/l, symptoms of vitamin C insufficiency appear such as fatigue and/or irritability, and concentrations under 11 μmol/l result in a condition known as Scurvy^22^.

Although uncommon, side effects of vitamin C supplementation have been reported in the literature. These possible adverse effects depend on dosage, route of intake, and frequency and duration of vitamin C supplementation^22,148^. Doses greater than 1000 mg per day may cause diarrhea, abdominal bloating, and kidney stones. However, from the short-term use of vitamin C, no significant adverse effects have been reported, especially for high doses up to 200 mg/kg/day (15 g per day) and even for the higher reported dosages of 1500 mg/kg three times per week (100 g of vitamin C) in cancer patients^2^. From a survey of 9328 patients who used high-dose intravenous vitamin C for 12 months, only a small percentage (101 side effects), most of them minor, were reported including lethargy/fatigue in 59 patients, change in mental status in 21 patients, and vein irritation/phlebitis in six patients^148^. The authors in the studies of our review have not reported any complications or AEs attributed to vitamin C therapy, neither solely, nor in combination with other antioxidants.

### Study limitations

By design, our narrative literature review is not systematic and follows no specified standards or protocols, nor a relevant meta-analysis is conducted. Although this type of review has inherent limitations in terms of objectivity, completeness of literature search, and interpretation of findings, as it is much more prone to selection and other biases, we tried to our best to be objective and arrive at a comprehensive understanding of the state of the science related to the problem. Even though our review is evidence-based, as a narrative review, is not always considered massively helpful in terms of the scientific evidence it brings. A future systematic review of RCTs and a meta-analysis could definitely address more comprehensively the postoperative administration of vitamin C in CS patients.

## Conclusion

In summary, our narrative review identified a number of prospective cohort studies and RCTs which show that vitamin C is a safe and affordable supplementation therapy that may help in various patients who are undergoing CS or other cardiac procedures. Vitamin C reduces the risk of significant cardiovascular complications and costs, both during the hospital stay and potentially for up to the first 1–2 months after surgery. This is particularly beneficial because remodeling and restoration of the heart’s muscle and nerve tissue occur during the same period of time.

Several research questions remain under consideration. Should vitamin C be administered routinely in CS patients? Has vitamin C a place in CS perioperative and postoperative optimal therapy? Which patient population could benefit most from this regimen? For how long, in which dose and by which route should it be administered? Should vitamin C be administered solely or in a combination with other antioxidants and/or other medications? Future investigations need to address such scientific questions through more sophisticated RTCs, preferably organized as multicenter studies, adequately powered, well stratified, and blinded, in order to enlighten the present knowledge gap about the use of vitamin C postoperatively, following CS. A postoperative vitamin C supplementation study that extends beyond the first 5–7 postoperative days, namely during the period of tissue remodeling, up to 4–6 weeks postoperatively, has not been conducted so far. Therefore, the potential of possible clinical benefits from such an intervention remains unclear. Taking into consideration the current evidence, one can hypothesize, that vitamin C supplementation for this longer period might benefit CS patients in ways that have not been previously reported in the scientific literature. This potentially paves the way for improvements in the therapeutic strategy for certain groups of CS patients, and subsequently a more efficient clinical approach.

## Ethical approval

This is a narrative literature review manuscript. Ethical approval is not applicable to this review.

## Consent

This is a narrative literature review manuscript. Informed written consent is not applicable to this review.

## Sources of funding

No funding was used for conducting the current review.

## Author contribution

A.A.: conceived of the idea for the study; A.A., E.S.S., and K.A.: designed the methodology; A.A.: searched the databases; A.A., M.C., and T.A.: reviewed the scientific articles; A.A. and M.C.: analyzed the data; A.A. and T.A.: created the tables; A.A.: wrote the first draft of the manuscript. All authors contributed to subsequent versions of the manuscript. All authors read and approved of the final manuscript.

## Conflicts of interest disclosure

The authors declare that they have no conflict of interest with respect to the current study.

## Research registration unique identifying number (UIN)

This is a narrative literature review manuscript. Research registration unique identifying number (UIN) is not applicable to this review.

## Guarantor

Athanasios Athanasiou, M.D., M.Sc., M.P.A., Department of Cardiothoracic Surgery, Nicosia General Hospital, Nicosia, Cyprus.

Elpidoforos S. Soteriades M.D., ScD Environmental and Occupational Medicine and Epidemiology, Department of Environmental Health, Harvard T.H. Chan School of Public Health, Boston, MA,02115, USA Healthcare Management Program, School of Economics and Management, Open University of Cyprus, Nicosia, 2220, Cyprus.

## Data availability statement

This is a narrative literature review manuscript. Data sharing is not applicable to this article (review).

## Provenance and peer review

This paper was not invited.
